# Spirit of the British and American Periodicals

**Published:** 1841-07-01

**Authors:** 


### Spirit of the British and American Periodicals &c.

### On the Diagnosis of Abdominal Inflammations. By W. Griffin, M.D., &c.*

Dr. Griffin has on a former occasion directed attention to the fact, that inflammatory, or other affections of the spinal cord, or of its nerves, at their origin, more frequently simulate abdominal and thoracic inflammations, and are more frequently mistaken and maltreated, than is at all imagined.

He reasons thus. There are three effects very common to inflammation, or disorder in the spinal cord, or at the trunks of its nerves. 1st, superficial tenderness, more or less exquisite, and either limited to the integument immediately over or about the affected portion of the cord, or extending thence to the front of the abdomen or thorax, in the direction of the spinal nerves; or occupying the whole cutaneous surface of those parts of the body, which are below the portion of the cord affected. 2ndly, pain either close to the affected portion of the cord, or at the extremities of the nerves, which have their origin there; or in the ganglionic nerves supplying the viscera, which have connexion with that portion of the cord. 3rdly, loss of power evinced in partial or complete palsy

* Dublin Journal, May, 1841.

of the parts or organs, to which the affected nerves are distributed. These effects often occur simultaneously, but any one of them may also occur independently of the existence of the others, offering very strong evidence, that the sensibility of the surface or skin, and that which exists in internal organs, is dependent upon nerves, which, though sentient, are as distinct from one another, as they are from those on which the power of motion depends. Keeping these ordinary effects of disorder of the spinal cord in view, it must appear obvious, when we detect soreness or tenderness on pressure, in the region of the liver or spleen, or of the lower abdominal viscera, how important and essential it is for us to ascertain whether the soreness be superficial or deep seated, which we can almost always do by careful examination. Again, when pain is complained of in the region of the liver, or middle or lower parts of the abdomen, how necessary is it to ascertain, whether, as in the case of soreness, it be superficial; and, if deep seated, whether it be merely an affection of the nerves of the part, and probably connected with some affection of the adjoining portion of the spinal cord, or whether the internal organ itself be in a state of acute or chronic inflammation. Finally, if there be oppression in breathing, whether it arises from deficient action in the respiratory nerves, and, as a consequence, imperfect action of the respiratory muscles, or imperfect performance of the process of oxygenation of the blood in the lungs; or from actual inflammation, or organic disease of the mucous membrane, or parenchyma of these organs.—Or if there be obstinate constipation of bowels, whether it depends on spasm, or enteric inflammation; or whether purely on deficient power, or partial palsy of the muscular fibres of the intestines.

Dr. Griffin relates two highly interesting cases, and follows them up with this conclusion,—*that,*

In determining the diagnosis of abdominal inflammation, where both pain and tenderness on pressure exist, we should always endeavour to ascertain:

1st. Whether there be any pain or tenderness on pressure in the corresponding portion of the spinal column; because, if there be, although it may not absolutely decide, whether inflammation be present or not; it is quite sufficient to account for both the pain and tenderness, without assuming the existence of any inflammation.

2ndly. Whether if there be no spinal tenderness or pain, the soreness of the abdomen be superficial or deep seated, which may be ascertained with tolerable certainty in all cases by an examination directed to that end. And whether, if both superficial and deep-seated, as it usually is, in peritoneal inflammation, gentle, steady pressure with the flat of the hand can be easier borne, than with the points of the fingers. In pain and soreness from affection of the spinal nerves it commonly can be so borne, while in peritonitis every kind of pressure, and even the weight of the bed-clothes, is very distressing.

3rdly. Whether the boundaries of the pain or soreness extend beyond what the suspected inflammation could produce. Thus, if inflammation of the liver be suspected, and we find the soreness extending to the spine of the ilium, or groin, or to the opposite side of the abdomen to which the liver does not extend, it is obvious, the soreness cannot be attributable to mere disease of that organ. Again, if the whole abdomen be tender to the touch in a case otherwise closely resembling peritonitis, and we find the tenderness is not confined to the abdomen, but extends over the hips and lower extremities, it is obvious, we can attach no importance to the abdominal soreness as a sign of inflammation.

Finally, it should be recollected that constipation may depend on mere loss of power in the intestinal nerves, as well as on spasm, obstruction, or inflammation, as the treatment in each case must necessarily be modified, or directed by the supposed cause of this symptom.

---

### Is the Representative System adapted for Medical Institutions? *

The following are the opinions of our Dublin contemporary on this head:—

"We do not profess to be politicians, or to deal with the question of representation, either in parliament, or in municipal corporations; but *of this we are certain, that the representative system is not at all calculated for medical institutions.* To say that the system would be a mere experiment, is comparatively a trivial objection; every anticipation must be as to the probability of direct mischief arising from such a proceeding. At present there is unfortunately but too much discord and display of bad feeling in our profession; but, as yet, party spirit and politics have had little or nothing to do with the matter. Introduce, however, the representative system, and every town in the empire, (in Ireland at least,) will be the scene of medico-political anarchy, and the far-lauded "Faculty" will merely be the arena for the medical demagogue; for it requires but little penetration to foresee, that impudence and blustering, and not professional eminence, would be the passport to a seat in the new assembly. Politics and medical reputation are in general heterogeneous articles, *and the politician is rarely a skilful, or, at least, an employed practitioner;* yet it is precisely such a character that would qualify him, under the representative system. If medical men are to steer clear of politics, and this seems to be agreed on, let them have nothing to do with representatives, and being represented. The best judges view elections in medical institutions, although sometimes necessary, as necessary *evils;* and consider, that the fewer they are, the better for the welfare of the establishment."

---

### Two Cases of Glanders in the Human Subject.†

We are induced to notice these two cases, because it would appear that the symptoms resulting from inoculation of the human subject with glandered matter differ materially in different instances, and an acquaintance with those differences is requisite. Many persons seem to imagine that a set of distinct and specific symptoms exist, and that any departure from the standard is, pro tanto, evidence that the complaint is not glanders.

### Case 1.—*Report of a Case, by J. B. Tytler, Esq.*

Charles Higgs, a stout young cab-driver was admitted into the Westminster Hospital on the 16th June, having been ill for five weeks, with severe rheumatism, pains of the legs and arms, increased at night, but without redness or swelling of the joints; the tongue furred in the centre; pulse very frequent; bowels rather confined. About three weeks ago he was bled and blistered, and felt some relief. He had three several abscesses in the lower extremities. He was treated by warm bathing, diaphoretics, and regulation of the bowels, and appeared to be making some progress toward recovery, until 23d June, when he complained of severe pain in the left leg, and swelling of the knee, with muscular pains of the chest unaccompanied by any marked constitutional symptoms. Relief was obtained from mustard cataplasms to the knee, and he continued under the former treatment, without marked alteration, till the 29th. He complains of violent pain in the head, and has had a rigor, followed by heat and fever. Ordered eight leeches to the nape of the neck, and saline purgatives, with antimony, every six hours.

---

* Dublin Journal, Jan. 1841.

† Edinburgh Monthly Journal, June, 1841.

July 2d. He has again experienced some relief: the head has been shaved, and is easier; some patches of inflammation appear on the scalp. Ordered to continue the saline purgatives.

4th. An extensive but very flat carbuncle has formed on the vertex, and having been freely incised, is dressed with resinous ointment, and poulticed. There is considerable fever.

5th. Another carbuncle has formed on the left eye-brow. The right eye-lid and cheek are much swollen and cracked in several places, discharging an extremely fetid sanies. The scalp looks erysipelatous, and is dusted with flour, and covered with cotton wool. The pulse is quick and weak; and the breathing hurried. He complains of sore throat. He takes wine and cinchona.

6th. The throat is now extremely sore: the tongue, which had been usually pretty moist, is parched and red; breath fetid; some offensive discharge from the right nostril; pulse very rapid and small. On examining the throat, many irregular yellow ulcerated patches are seen. Wine and bark continued.

7th. A number of pustules have appeared on the arms and legs; the throat is more ulcerated, and the right eye closed by the swelling; the sanious discharge continues. He has fallen into a low muttering delirium.

8th. All the symptoms are aggravated; and his occupation having given rise to suspicions of glanders, which were confirmed by persons who had seen the disease in the human subject, his friends were minutely examined, and now acknowledged that not only had he been working about a glandered horse, but that a fellow-servant had died recently from the disease, by infection from the same animal. The horse also died. The patient himself died at 3, a. m. On examination 10 hours after death, the fauces were found coated with sanious matter, which had collected about the angle of the jaws, and flowed into the trachea and gullet. The palate and fauces, with the posterior nares were sloughy; a small ulcerated patch existed in the lining membrane of the trachea, just opposite the cricoid cartilage, and the larynx was generally much inflamed. The thoracic and abdominal viscera were healthy; the brain was not examined.

This case seems to have lasted long, and, for forty-eight days, the symptoms were very ill-defined.

CASE 2.—*Report of a Case of Glanders in the Human Subject. By Alexander Graham, Esq.*

"J. S. a carter, aged seventeen, consulted me on the 2nd February 1840, about a pain he had felt for some days previous in the index finger of the right hand, which was swelled, and slightly inflamed. The skin covering the first phalanx was of a livid colour, and there was fluctuation underneath. He had a considerable degree of febrile irritation. On opening the finger, a very small quantity of thin greyish-coloured fluid escaped, without affording any relief from pain. The soft parts appeared dead, but no line of separation was observable, nor indeed for many days after were there any symptoms of impending danger. He continued in nearly the same state till the evening of the fifth day from the time I first saw him, when the soft parts covering the first phalanx came off during the dressing, leaving the bone exposed. On removing the bone, which was easily done, without causing the least pain to the patient, the part presented the appearance of a very healthy-looking sore, and the redness and swelling of the hand and finger entirely disappeared. On the following day, however, a new and more alarming train of phenomena presented themselves. During the preceding night, he experinced a great tendency to rigors; there was increased pyrexia, and he complained of a pain over the spine of the left tibia, near its distal extremity. At this place there was a small circumscribed tumor, which was very painful to the touch. The integuments were slightly inflamed, and an obscure fluctuation was also observed. On interrogating him and his friends closely regarding the sore on his finger, it was discovered that he had been driving a glandered horse, and that it was possible it might have been produced by his finger, which was scratched, coming into contact with the diseased animal.

On the eighth day the tumor over the spine of the tibia had nearly disappeared, but he experienced severe and constant pain from the site of the swelling along the inside of his leg, as far as the middle of the thigh, which was tense and swelled. He shewed much restlessness and anxiety; his stools were of a darkish-green color, thin, and fetid; his tongue was loaded, and he had great thirst. The pulse was quick and small.

On the following day (the ninth) his finger appeared to be healing; but he informed me that he had passed a very restless night, without sleep, and that the pain of his leg was more severe. The absorbents now became red and hard, and their course could be traced distinctly as far as the knee joint. The leg and thigh were more swelled, and the tumor was hardly perceptible. The pulse was 120, and small, the skin hot and dry, and the tongue very foul.

Next day I found the leg and thigh more swelled, and found it necessary to make several incisions on the inside of the leg. They did not bleed much, but nevertheless afforded the patient some relief. His bowels were open, and the stools were fetid. From this time he became gradually worse; and on the evening of the day before that on which he died, (the twelfth), when I visited him in company of a professional friend, we found a general tumefaction of the whole body, but mostly of the left side. His head and face were much enlarged, and, in addition to this increased swelling, there were all over his body and face several distinct and prominent pustules, resembling variola, none of which were visible the preceding day. The inside of the leg and thigh were also covered with a great number of gangrenous spots, of irregular size and shape, ranging from the bulk of a split pea to that of a shilling, but without vesication, or the least appearance of separation. These gradually increased in size and number until he died, on the 13th February.

During the whole course of his disease, and even up to the very close of it, there was not the least manifestation of any disturbance in the sensorial functions."

---

### Re-union of a Separated Portion of Finger. By A. Graham, Esq. Falkirk.*

A joiner of middle age, and apparently healthy constitution, while splitting wood with an axe, cut through the index finger of his left hand, between the first and second phalanges. He lifted the separated part from among the shavings, and immediately walked a few yards to a place where Mr. Graham happened to be. Being asked for the amputated portion, he took it from his waistcoat pocket, and laid it on the table. Mr. Graham fixed it on by two sutures, and adhesive strap, and on the fourth or fifth day, a pair of scissors being applied to the point of the finger, he distinctly felt them. Complete union took place, with restoration of the powers of the part which had been separated.

---

### Cases and Observations on Diabetes. By Robert Christison, M.D. &c. &c.†

A long and valuable paper is contributed to our Scottish contemporary by Dr.

---

* Edin. Monthly Journal, April, 1841. † Ibid.

Christison. He relates four cases. We have not space for these, and shall merely extract such observations as may strike us as instructive.

1. *Sugar not always discoverable in Diabetic Blood.*—The first case, says Dr. C., is a good example in support of the statement lately advanced by Dr. Macgregor, that in diabetes the urea is frequently not defective, as previous experiments seemed always to indicate, but is even sometimes absolutely in excess. It is at variance, on the other hand, with an equally important statement by the same gentleman, that sugar may be always detected in the blood. The search for sugar was made in this instance with very great care, and by a test which I have found capable of detecting a single grain of diabetic sugar in a thousand grains of the densest healthy urine. Yet I may say, that not a trace was indicated; for the trivial evolution of gas might have come from the water, or from the yeast itself! On another occasion, about eighteen months before, I was not more successful. It would appear, then, that the presence of sugar in the blood is not a general fact, and therefore can scarce form a necessary step in those functional derangements which constitute the pathology of diabetes.

2. *Voracious Appetite not constant.*—This patient, when put on animal diet, consumed less real nutriment than is contained in the diet which has been found necessary for prisoners not subjected to hard work, and which amounts on an average to twenty-five ounces, partly vegetable and partly animal.

3. *Strictly Animal Diet requisite.*—If a sensible amelioration is to be looked for with any confidence, the injunctions of Rollo and his imitators, to enforce a rigorous animal diet, must often be faithfully followed. This object it is by no means so difficult to accomplish as might be thought, provided some variety be allowed, and articles be avoided which are hard of digestion, or abound too much in fat. In the present case no very decided amendment was observed for two months under a general diet; but under a pure animal diet, which, so long as the man was under my notice, seemed to be followed by him without reluctance and with fidelity, the characters of the urine improved essentially in a few days; and the improvement was afterwards steadily maintained.

4. *Quantity of Solids in the Urine to be rigorously determined.*—This appears the only true criterion of the progress of a case. The quantity of the urine taken singly is a fallacious criterion, yet it is often trusted to in practice. Though reduced in quantity, the urine may be increased in density; and in that case there may be no real amendment in the essential character of the disease,—the preternatural discharge of solids by this secretion. The quantity and density ought always to be taken together. For greater accuracy, it is well to calculate the total solids from these data, by the following formula. If D′ represent the difference between the density of the urine and that of water, and Q the number of ounces discharged in twenty-four hours, the daily discharge of solids by the urine is $QD' \times 0.00233$.—Libr. of Prac. Medicine, iv. 248.

5. *Mode of ascertaining the Presence of Urea.*—In commenting on Case 2, Dr. Christison points out the abundance of urea in the urine, and proceeds:— For more than ten years I have been persuaded of its frequent presence, and have repeatedly proved its existence during my clinical lectures. During that period I have never failed to detect it. As at least five-and-twenty cases must have been examined by me in the interval, and on two recent occasions I succeeded in finding it where others had failed, it seems not improbable that urea is always present. The plan I have followed in searching for it, is to concentrate the urine, when quite recent, over the vapour-bath to an eighth, but not farther, and then to add an equal volume of nitric acid previously diluted with its own weight of water, and allowed to cool. Sometimes the nitrate of urea does not form for a few hours, or not till during the night, to the cold of which the mixture ought always to be exposed before we can venture to conclude, from the absence of crystallization, that urea is wanting. I have repeatedly known the nitric acid fail to act where the urine had been evaporated to dryness, or to a thick syrup, and then redissolved, even though the evaporation had been conducted in the vapour-bath. Although I am inclined to think that urea is always present, it is by no means always in excess, as in this and the preceding case. On the contrary, as will be seen from the fourth case, it is sometimes very defective.

6. *Laxity with regard to the Determination of Diabetes Mellitus. Precautions necessary.*—"The opinions which prevail at present as to the sanability of *diabetes mellitus* are contradictory. A few authors maintain, that, although very untractable, the disease may be cured in a reasonable proportion of cases: but the greater number of recent authorities state, as the result of their experience, that it is sooner or later, but inevitably, mortal; and my own observation hitherto has corresponded with that doctrine. The discrepancy may be in part explained in two ways. In the first place, some practitioners seem much too easily satisfied as to the evidence required to establish a cure of a case of diabetes. If they succeed in reducing the quantity of urine to three pints daily, in lowering its density to 1030, in removing the saccharine taste, and in restoring, as they will have it, the urea, a recovery is supposed to be accomplished; the patient is dismissed, probably from an hospital; and because his subsequent fate is never heard of, he is considered to have sustained a radical cure. I need not say how fallacious the last circumstance often proves. What should be particularly considered, however, is, that sugar may be abundantly present in such urine as that now described, and that it cannot justly be inferred to be wanting, unless the urine resist the test of fermentation with yeast about the temperature of 80°. But secondly, it may be strongly suspected that some reported cures have from first to last been merely cases of *diabetes insipidus*. It is difficult to account otherwise for the density of the urine being repeatedly stated so low as 1020, 1015, and even 1010. I will not deny that such a state of the urine is possible in true saccharine diabetes. I can only say that I have never but once seen it so low in density as 1021; and this was after the patient had been long under a regulated animal diet, along with opium. Such being the fact, I cannot but entertain doubts as to the genuine nature of a case of alleged *diabetes mellitus* with the urine of very low density, so long as absolute evidence is not furnished of sugar being present; and in some of the cases alluded to, reliance has been placed entirely on the taste of the urine, its odour, and the appearance of its extract, which are all fallacious criterions. I have met lately with two instances where the nature of the symptoms was thus for some time misunderstood. In one of them, a woman who was thought to be diabetic because the urine flowed profusely and seemed to have a sweet taste, its density proved to be only 1010. Being consequently led to doubt the soundness of the opinion previously formed as to her complaints, I proceeded to test the urine with yeast, and found that it did not contain a particle of sugar. The other case was that of a gentleman not many weeks ill, who presented all the general signs of saccharine diabetes, and passed a large quantity of urine which was thought to possess a sweetish taste and odour. I confess that at first, so long as my attention was confined to the general symptoms and sensible qualities of the urine, I felt inclined to concur with the opinion entertained that this was a genuine case of *diabetes mellitus*. But on examining the urine with care, I found its density was only 1004.5; urea was present in a proportion commensurate with its low density; yeast did not cause any evolution of carbonic acid at 80°; and nitric acid, digested for some hours with the urine in a moderate state of concentration, did not produce any oxalic acid. Both of these cases, then, were examples of *diabetes insipidus*, and as such were much more susceptible of cure."

At the same time, Dr. Christison is far from disputing the possibility of cure, if the treatment be commenced at an early period.

7. *Case* III. was a fatal one, granular disease of the kidney supervening. On this hint Dr. Christison speaks. At a time, says he, when the characters of diabetes were all accumulated in their severest form, the disease underwent a total change. The diabetic properties of the urine by degrees vanished; characters the very opposite began to present themselves, and the state peculiar to granular disease of the kidneys was soon established; the patient lingered in this condition for a long period, the urine being for some months about the healthy average in quantity, 1010 in density, albuminous, and entirely without sugar; death ensued from obstinate diarrhœa, one of the secondary affections most frequently observed in Edinburgh during granular disease of the kidneys; and in the dead body dropsical effusion into the cavities, another secondary affection, was also found, together with extensive granular degeneration of the renal textures. It is well known that, as death draws nigh in saccharine diabetes, the urine for a few days before death ceases to contain sugar, and returns nearly to its healthy state. But here is an instance where for several months before death, and to all appearance without any connexion with the treatment, all the remarkable properties of the urine which characterise diabetes, were completely and permanently removed. The argument drawn from the former class of facts, in favour of this disease being functional only, so far as concerns the affection of the kidneys, is thus, he thinks, not a little strengthened. "It is well to add," he continues, "that on several other occasions I have observed diabetic urine more or less albuminous. In one of these, the impregnation with albumen was considerable, and the kidneys were found after death in an advanced state of granular degeneration. But this case was not under my own care, and I cannot now obtain an account of it."

8. *Rapidly Fatal Diabetes—Small Amount of Urea.*—"It is seldom," observes Dr. C., "that the disease runs its full course, not curtailed by some incidental acute disorder, in the short space of eighteen months, which seems to have been the extreme limit here. The case is obviously a remarkable instance of that kind which has supplied some authors with a basis for the doctrine, that diabetes mellitus originates in disorder of the functions of the stomach and intestines. It commenced abruptly after an acute affection of that nature. Such occurrences, however, are rare. I have paid particular attention to this point for some years past; and every case I have myself attended for the last seven years was carefully ascertained to have begun without any precursory derangement of digestion. The characters of the urine corresponded with what I have called the acute nature of the attack; and departed more from the healthy state than in any instance I have examined before or since. I suspect that all along the urea must have been very greatly reduced, not merely in its proportion to the other solids, but likewise in its total daily amount. It was so at least towards the close, even at a time when the urine showed a tendency to return to its healthy condition; for on the twentieth and sixteenth days before death, its total amount was only 294 and 130 grains in twenty-four hours, the higher of which sums is a trifle more than half the average of full health in a robust well-fed man. I had never previously met with an instance in which sugar of a snow-white colour, and nearly pure, could be so easily obtained by simple evaporation.* The rapid

* I have recently met with a similar instance, which also, like that detailed above, commenced abruptly, and has presented an unusually acute character.

change which often takes place in the properties of the urine before death in diabetes, when unmodified by incidental diseases, is here well exemplified. In a few days the urine fell from 320 to 32 ounces; its density also sunk; and its urea became so abundant, as to exceed the daily discharge in a state of health, being on one occasion so high as 754 grains. On the 11th March I found the urine characteristically diabetic, as usual. When next day, in the absence of Dr. Peebles, his clinical clerk showed me amber-coloured urine, and reported that it had fallen in amount to a few pints, I felt assured from previous experience, that the patient, though to appearance more comfortable, and pleased with the change, was going to sink; and such was the speedy result."

We should observe that the patient died with tubercular deposits and excavations in the lungs.

---

Vaccine Crusts the most Effectual Packages for the Preservation of the Vaccine Virus. By John Epps, M.D., Director of the Royal Jennerian and London Vaccine Institution.

"The vaccine pock is similar to the *vitreous* humour occupying the largest portion of the chamber of the eye. It is a compages of vessels with their contained fluid. The *aqueous* humour of the eye escapes by a single puncture, like the virus of the vaccine pock: hence the indurated pock, the scab or crust of the vaccine pock, contains the dried matter in its cells, which, being broken down, and moistened with the wetted point of the lancet, has been found effective in hot climates, when attempts to preserve the vaccine ichor in other forms have failed. The crusts or scabs we have been able to collect in this country, we learn, by letters from the East and West Indies, have withstood the heat of their vertical sun, and spread protection through the plantations, and the surrounding districts.

"N.B.—These crusts, when levigated and moistened, and worked into a fine pulp, have been used by many practitioners in tropical climates with the most signal success. Mr. Johnston, the late secretary of the institution, was the first who suggested this plan."—*Lancet, March* 13, 1841.

---

Dr. Guerin on the General Characters of Rachitis.

Mr. Bigger, Surgeon to the Adelaide Hospital, has translated a valuable memoir of Guerin's for our contemporary of Dublin.* The memoir is long and will richly repay perusal. We must content ourselves with the general conclusions at which M. Guerin has arrived. They are as follows:—

1st. Rachitis is a general or constitutional disease of childhood, characterized by the alteration or the perversion, or even the suspension of the work of development and of reparation in the organism, and principally in the osseous system.

2nd. The progress of rachitis, considered as an affection of the skeleton, in-

---

In this case the urine was 1035·5 in density, 218 ounces in daily quantity, and all but colourless. The daily discharge of urea was only 135 grains. When evaporated to the consistence of a moderately thick syrup, the residuum, under exposure for a few days to a cold of 32°, became a congeries of snow-white granular crystals.

* Dublin Journal, January, 1841.

cludes three distinct stages: that of *incubation* or *effusion*, of *deformation*, and of *resolution* or *eburnation*. To each of these periods, corresponding peculiar symptoms belong, and alterations peculiar to the osseous tissue.

3rd. The influence of rachitis on the osseous tissue is shewn by four distinct orders of facts; deformation, alteration of tissue, arrest of development, and retardation of ossification.

4th. Rachitic deformation of the skeleton is developed in succession, from below upwards, from the bones of the legs to the thighs, from the thighs to the pelvis, then successively or simultaneously, the different parts of the upper extremities, the thorax, and lastly, the spinal column and the cranium. The degree of deformity accords with the order of development, whence it follows, that rachitic deformity of any portion of the skeleton implies always deformity of the parts situated below it.

5th. Most of the bones of the rachitic skeleton are always relatively less developed, in length and breadth, than the bones of the healthy skeleton; this reduction, which is independent of deformity, still follows the same law: that is to say, successively from below upwards, and gradually from above downwards; the proportion, according to which all parts of the skeleton are abbreviated from below upwards, can be expressed by a regular series of numerals, which admits of a very close deduction of the dimensions of the whole skeleton, that of one bone being known.

6th. The maximum reduction of the lower extremities, compared with that of the upper, establishes between them relations of length, which repeat and perpetuate those of the age when the disease was first developed.

7th. The reduction of the bones in rachitic adults is the compound result of arrest of development in the osseous system, under the direct influence of the disease, and of retardation of growth when the disease has ceased.

8th. The texture of rachitic bones presents characters totally different, according as they are observed during each of the three stages, or at the beginning or end of each stage, or according to the chronicity or intensity of the affection.

9th. During the stage of incubation of rachitis, an effusion of sanguinolent matter occurs into all the interstices of the osseous tissue, into the cells of the spongy tissue, the medullary canal, between the periosteum and the bone, between the concentric laminæ of the shaft, between the epiphyses and the shaft, between the points of ossification in the ephysis and their cells, in the short and flat bones, in a word, in all parts of the skeleton, and in all points of the osseous tissue, where branches of the nutritive vessels are distributed; from this effusion results the duplication of the parts composing the tissue, and the swelling and puffing out of different portions of the skeleton.

10th. During the second stage of rachitis, (deformation,) whilst the web-work of the osseous tissue loses its consistence and becomes softer, the matter which continues to infiltrate all the interstices of the bony tissue begins to become organized; it passes successively from the cellulo-vascular, to the cellulo-spongy form; this adventitious matter abounds principally between the periosteum and the bone, between the medullary membrane and the innermost bony layer, and in the short and flat bones, between the periosteum and the bone, and between all the laminæ.

11th. During the third stage, (resolution,) the adventitious tissue in the long bones, and in some of the short and flat bones, passes into the state of compact tissue, and appears to become confounded with the old tissue which recovers its former hardness; this addition of a new tissue to the old gives a very great degree of density, and above all, a very great breadth to some parts of the bones, which were the seat of the organization of new spongy tissue in the preceding period.

12th. In that state which I have denominated rachitic consumption, and which results from an exaggerated degree of the affection, the duplication and distancing of the parts composing the osseous tissue has been such, that their union never takes place, and the organization of the effusion does not occur. In this case the osseous partitions and laminæ remain apart, and the consistence of the primitive bone is so much reduced, that there sometimes only remains a thin shell of the external part of the bone.

13th. The texture of rachitic bones in adults, when the disease has ended, presents a compactness and hardness, greater than in the natural state. This is called rachitic *eburnation*, when there is no longer any means of distinguishing the traces of union between the old and the new bone.

14th. Those deformities of the spine, which happen towards the age of puberty, and all those which have not been preceded by deformity of the legs, are not of a rachitic nature.

15th. Rachitis is an affection essentially different from *scrofula* or from *tubercular affection of the bones*, as well as from *all kinds of ramollissement which occurs in adults*; for these latter, the term *osteomalacie* has been exclusively reserved.

---

### Compression of the Heart.*

Mr. Adams brought this case before the Pathological Society of Dublin.

Patrick Devine, aged twenty-six, admitted into the Whitworth Hospital, for an attack of chronic bronchitis, accompanied by empyema. The disease did not yield to treatment, although active measures were employed; the cough was still severe, and the quantity of fluid in the chest seemed increasing. After some time he was exposed to cold and got much worse; the dyspnœa increased, accompanied by lividity of face, swelling of the jugular veins and inability to expectorate. About this period also he was attacked with erysipelas of the right arm, on which a large gangrenous spot subsequently made its appearance. On the 19th day after admission Mr. Adams found him sitting up in bed, gasping for breath, holding by the sides of his bedstead; his eye-balls protruded, his face livid and bathed in a cold perspiration. A redish serum was flowing from his mouth, and his respiration was inaudible, except at the upper part of one lung. His belly was swollen and tympanitic. He remained for about eighteen hours in this state, and died the following night. On examination after death, both pleuræ, but particularly the left one, were filled with bloody serum. The left lung was adherent to the posterior part of the pleura, compressed and carnified. The respiratory process seemed to be carried on wholly by the upper third of the right lung. The heart lay compressed and quite horizontal in its direction, the right ventricle folded on itself, and the whole organ compressed and flattened. Mr. Adams here exhibited the heart, the right ventricle of which, as well as the pulmonary artery, was quite empty, the ventricle itself being thrown into three rounded folds. This was the chief peculiarity of the case. Mr. Adams then exhibited the preparation, as also a cast taken from it, and observed, that this was a condition of the heart he had never before witnessed. He also remarked that the appearance of the parts would lead any one who examined them to infer that this condition was not one of recent occurrence. The parts of the heart which lay close to each other in the line of the folds were firm and of a parboiled aspect, shewing that the folds were of some standing. There was one symptom observed in this patient, viz., intermission of the pulse. How far this condition of the heart might explain it, or how far it was connected with the tympanitic state of the abdomen, Mr. Adams could not say, but he had frequently observed intermission of the pulse in connexion with abdominal tympa-

---

* Dublin Journal, May, 1841.

nites. He was inclined, however, to think that for some time before death the right ventricle and the pulmonary artery had ceased to perform any functions.

---

### CURE OF SPINA BIFIDA.*

Sir Philip Crampton brought this subject before the Society. He alluded to Sir A. Cooper's paper on the treatment of the disorder in the second volume of the Medico-Chirurgical Transactions. Sir Astley then detailed the treatment and results of three cases. The first was that of an infant two days old, in which he found a small tumour of the lumbar region, capable of being obliterated by pressure, the whole of the fluid passing into the vertebral canal. It immediately occurred to him, that if the fluid could be kept in, while the process of consolidation of the bones of the spine went on, a cure might be effected. Having placed the child in a prone position, he made a cast of plaster of Paris, adapted for making due pressure on the tumour, and secured this by proper bandages. He afterwards substituted for this a pad, from which much less inconvenience was experienced. At the age of eight years the child was running about and in good health, still wearing the pad, but not cured. The next case was one in which he attempted to confine the fluid by means of pressure, but having failed in this, he had recourse to a plan of treatment founded on a principle introduced by Mr. Abernethy, in the treatment of psoas abscess. He made a small puncture in the tumour, and having evacuated a portion of the fluid, he then closed the aperture with adhesive plaster. This was done ten or fifteen times, and at each operation a quantity of fluid removed. This child ultimately recovered, the bones of the spine having closed, leaving behind a loose moveable sac, and when inspected by Drs. Marcet and Yellowly some years afterwards, was found to be quite well. This, however, was the only successful case which occurred in Sir A. Cooper's practice. Another case of the same kind occurred in Sir Philip Crampton's practice. The operation of evacuating the tumour according to the mode employed by Sir A. Cooper, was performed twenty-two times, with some local irritation, but without any unfavorable or threatening symptoms. The sac gradually thickened, the consolidation of the bones went on, and in the course of seven months the cure was completed. The child is at present in good health, but has, as in Sir A. Cooper's case, a loose bag on its back. With respect to the spontaneous cure of the disease, Sir P. Crampton was not aware of any instance on record. In general the patients die at a very early age. He felt, therefore, extremely happy in having an opportunity of exhibiting to the Society an example of spontaneous cure in the person of a gentleman who had been recently under his care for another complaint. While examining his chest, Sir Philip Crampton detected the remains of the sac, originally the result of spina bifida, and as he expressed a wish to shew it to his professional brethren, the gentleman had in the kindest manner consented to come before the meeting, and exhibit the tumour. Sir Philip here shewed the sac, which extended over the lower dorsal and the lumbar vertebræ. The fissure in the vertebræ appeared to have been originally about three inches in length.

---

* Dublin Journal, May, 1841.

